# Analysis of the Clinical Effects of the Combination of Mycophenolate Mofetil with Either Tacrolimus or Cyclophosphamide

**DOI:** 10.6061/clinics/2020/e1820

**Published:** 2020-11-02

**Authors:** Xuebing Zhang, Pei Liu, Zhen Zhang

**Affiliations:** Department of Nephrology, Lanling County People's Hospital, Linyi, Shandong, China

**Keywords:** Mycophenolate Mofetil, Tacrolimus, Cyclophosphamide, Lupus Nephritis, Cystatin C, Transforming Growth Factor-1

## Abstract

**OBJECTIVES:**

Here, we aimed to compare the clinical effects of mycophenolate mofetil combined with either tacrolimus or with cyclophosphamide on lupus nephritis (LN) and to analyze their influence on the expression of cystatin C and on transforming growth factor-1 (TGF-β1).

**METHODS:**

A total of 234 patients were randomly divided into two groups: group A, for mycophenolate mofetil combined with tacrolimus (n=117) and group B, for mycophenolate mofetil combined with cyclophosphamide (n=117). The enzyme-linked immunosorbent assay was adopted to detect the expression levels of serum TGF-β1 and cystatin C before and after treatment.

**RESULTS:**

The total effectiveness rate in group A was much higher than that in group B. The times of effectiveness and effect validity in group A were much lower than those in group B. The expression levels of serum TGF-β1 and cystatin C decreased slightly after treatment in the two groups, and those of group A were much lower than those of group B.

**CONCLUSIONS:**

The combination of mycophenolate mofetil and tacrolimus showed better clinical efficacy on LN and was safer than that of mycophenolate mofetil and cyclophosphamide. Moreover, the drug combination of mycophenolate mofetil and tacrolimus greatly reduced the expression levels of serum TGF-β1 and cystatin C.

## INTRODUCTION

Lupus nephritis (LN) is a common complication of systemic lupus erythematosus. Approximately 10% of patients with LN will have end-stage renal disease, which is the main cause of death in these patients (1,2). The incidence rate of LN in China has gradually increased (3). Presently, the primary aim of the treatment of LN is to prevent the degeneration of renal function and to achieve its normalization (4), including an inductive (controlling immune-mediated inflammation through cytotoxicity and hormone therapy) and maintenance treatment (keeping LN at a low activity level through immunosuppression) (5). Moreover, mycophenolate mofetil is a common prescription drug used for the maintenance treatment (6). In clinical practice, cyclophosphamide is a common drug used for the inductive treatment of LN patients (7). Tacrolimus, which is a new immunosuppressive drug with powerful immunosuppressive action, can be used to treat all types of autoimmune diseases (8).

Cystatin C, whose level is less affected by renal extrinsic factors *in vivo*, is not only a non-glycosylated basic protein, but also a cysteine protease inhibitor with low molecular weight. It is only filtered and removed by glomeruli *in vivo*; thus, it can be used as an early marker of renal injury (9-11). Some studies have shown that cystatin C not only reflects the degree of renal injury in LN patients, but can also be used to evaluate the therapeutic effects and prognosis (12). Transforming growth factor-1 (TGF-β1) can be secreted by various cells, such as normal and malignant cells, in the human body. Moreover, bone tissues and blood platelets are its most abundant sources (13). TGF-β1, a cell factor expressed in the kidneys, is related to the development of tubulointerstitial fibrosis (14). Presently, there are only a few analyses on the influence of the combination of mycophenolate mofetil and either tacrolimus or cyclophosphamide on the serum levels of cystatin C and TGF-β1 when used for LN treatment.

In this study, the therapeutic effects of mycophenolate mofetil combined with either tacrolimus or cyclophosphamide on LN were compared and evaluated to discuss the influence of the combined treatment on the serum levels of TGF-β1 and cystatin C.

## MATERIALS AND METHODS

### General data

A total of 234 patients with LN diagnosed via renal biopsy in our hospital were enrolled in this study. The patients were randomly divided into two groups, i.e., group A, for mycophenolate mofetil combined with tacrolimus (n=117) and group B, for mycophenolate mofetil combined with cyclophosphamide (n=117). Furthermore, there were 53 men and 64 women in group A, with the pathological types including 29 cases of type III, 53 cases of type IV, and 35 cases of type V. Moreover, the average course of the disease was 11.65±4.57 months in group A. In group B, there were 48 men and 69 women, including 28 cases of type III, 56 cases of type IV, and 33 cases of type V. Further, the average course of the disease was 11.32±4.21 months.

The inclusion criteria included the fulfilment of the diagnostic criteria of LN, complete clinical data and good compliance, no medical history of epilepsy, and a good mental state and normal expression and comprehension abilities.

The exclusion criteria were other autoimmune diseases and renal lesions, pregnancy or history of surgery or trauma in the previous 3 months, allergy to drugs used in this study, and hemorrhagic diseases or coagulation disorders.

This study was approved by the ethics committee of Lanling County People's Hospital. All subjects agreed to participate in this study and signed a complete informed consent form.

### Treatment methods

Both groups received glucocorticoid pulse therapy. Initially, 0.5 g of methylprednisolone (Pfizer Manufacturing Belgium NV; Saudi Food and Drug Authority [SFDA] approval number, H20080284) was injected through an intravenous drip for 3 days. Then, oral prednisone acetate tablets (the Fourth Pharmaceutical Factory of Harbin Pharmaceutical Group; SFDA approval number, H23020185) were administered to the patients during the intermittent stage at a dose of 45 mg/day. The specific dose was adjusted according to the conditions of the patients.

The patients in group A received a multi-target treatment of mycophenolate (Roche Pharmaceutical Shanghai Co., Ltd., SFDA approval number: H20031240) and Tacrolimus (Zhejiang Hongsheng Pharmaceutical Co., Ltd., SFDA approval number: H20133162) during the 6-month induction period and the subsequent maintenance period. Furthermore, in the induction period, patients were also orally administered mycophenolate at 1 g per day and Tacrolimus at 4 mg twice per day. The initial doses of mycophenolate and Tacrolimus were adjusted to 0.75 g and 3 mg, respectively, if the patient had a body weight under 50 kg. Until the maintenance period, the doses of both drugs were reduced to 0.75 g and 3 mg respectively. The patients who achieved a complete remission during the persistent period were orally administered mycophenolate and Tacrolimus at 0.5 g and 2 mg, respectively, per day.

The patients in group B were administered cyclophosphamide (Jiangsu Hengrui Medicine Co., Ltd.; SFDA approval number, H32020857) through an intravenous drip (0.6 g once a week for 2 consecutive weeks). Simultaneously, mycophenolate mofetil tablets were administered at the same dose as that administered to group A patients. The treatment lasted for 6 months in the two groups. During the treatment, vital signs were monitored, and electrolyte and blood routine examinations were performed to detect problems and conduct a symptomatic treatment.

### Serum detection

A volume of 5 mL of venous blood was drawn from the subjects in the fasting state and was subjected to a 5-min centrifugation (4°C, 3,000 rpm) to separate the serum. Then, the supernatant liquor was obtained and cryopreserved in the refrigerator at -80°C for detection. The BS-1101 enzyme-mark analyzer, which was purchased from Beijing Linmao Technology Co., Ltd., was used to determine the expression levels of serum TGF-β1 and cystatin C using enzyme-linked immunosorbent assay (ELISA) kits. ELISA kits were provided by Shanghai Guduo Biotechnology Co., Ltd., with the product codes YM-S0090 and GD-QX0754. The blank hole (for which the same steps as those followed for the other two holes were conducted, aside from the addition of the conjugate reagent and samples), the tested sample hole, and the standard hole were set. For this, to the tested sample hole, 10 μL of sample was added to 40 μL of the diluent, and to the standard hole, 50 μL of each of the standard substances at different concentrations was added. Then, the reaction hole was sealed with a sealing membrane and placed in a water bath kettle at 37°C or in an incubator for 30 min, after which it was washed. Aside from the blank hole, to the other two holes, 50 μL of conjugate reagent was added, followed by incubation at 37°C for 30 min, and washing. Next, 50 μL of substrates A and B was added to each hole and these were kept away from the sun for 15 min at 37°C for color development. Then, 50 μL of stop solution was added to each hole. Moreover, the zero setting was used in the blank hole. The optical density of each hole was measured at the wavelength of 450 nm for 25 min. Finally, the serum TGF-β1 and cystatin C levels were calculated.

### Observation targets

The expression of TGF-β1 and cystatin C was observed before and after treatment in the two groups; the therapeutic effects and occurrence rates of adverse reactions were evaluated and the correlation between TGF-β1 and cystatin C was analyzed. The evaluation criteria of the therapeutic effects were the following (15): 1) complete remission (CR): the serum albumin and 24-h protein levels were in a healthy range after treatment, and the renal function was markedly improved; 2) partial remission (PR): the serum albumin and 24-h protein levels became normal after the treatment, but the renal function did not obviously improve; and 3) invalid: there was no significant change in the serum albumin and 24-h protein levels, and the renal function did not improve.

### Statistical analysis

SPSS 20.0 (IBM Corp, Armonk, NY, USA) was used for the statistical analysis, with the enumeration data represented as n (%). The chi-square test was used for comparison between the groups, with the measurement data represented as the mean±standard deviation (x±SD). The t-test was used for comparison between the groups, ANOVA was used for the comparison of the expression levels before and after treatment, and the Least-Significant Difference (LSD-t) test was used for back testing. The Pearson correlation coefficient was adopted for the bivariate normal distribution data. *p*<0.05 indicated a statistically significant difference.

## RESULTS

### Comparison of general data between the two groups

The general clinical data of the two groups were collected, as shown in Table 1. There were no significant differences in sex, age, body mass index, average course of disease, smoking and drinking habits, platelet and hemoglobin count, and pathological type, among other parameters, between the two groups (*p*>0.05).

### Comparison of therapeutic effects between the two groups

According to the statistical data of the two groups, the effectiveness rate of group A was much higher than that of group B, which indicated a statistically significant difference, as shown in Table 2 (*p*<0.05).

### Comparison of effective time between the two groups

**Figure 1:**
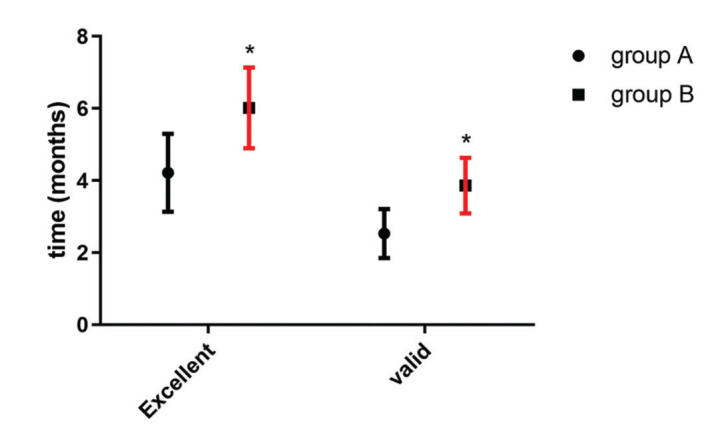
**Comparison of the effectiveness times between the two groups.** The results of the time of effectiveness and time of effect validity in the two groups indicated that these are much lower for group A than for group B, showing a statistically significant difference (t=12.510 *versus* t=14.000, *p*<0.001). Note: * represents the comparison with group A, *p<0.05.

As shown in Figure 1, the time of effectiveness and time of effect validity of group A were much lower than those of group B, showing a statistically significant difference (t=12.510 *versus* t=14.000, *p*<0.001).

### Comparison of the expression levels of serum TGF-β1 and cystatin C between the two groups

**Figure 2:**
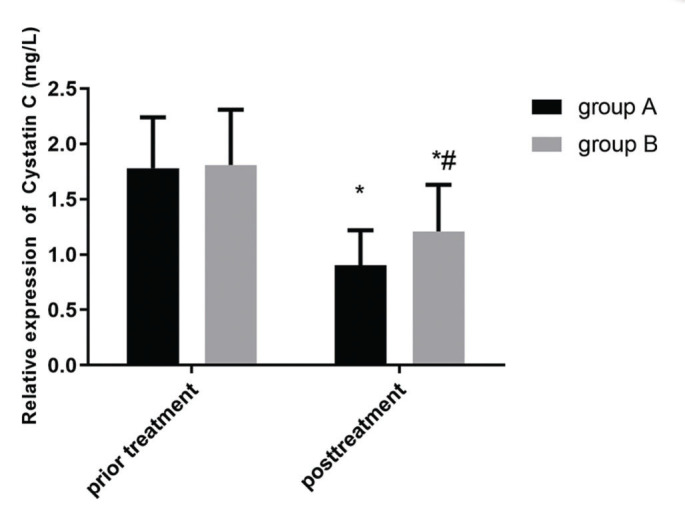
**Comparison of the serum levels of cystatin C between the two groups.** There is no significant difference in the expression level of serum cystatin C before treatment between the two groups (*p*>0.05). The expression level of serum cystatin C slightly decreased after treatment in the two groups, which indicated a statistically significant difference (*p*<0.05). Further, the expression level of serum cystatin C in group A is much lower than that in group B after treatment, which indicated a statistically significant difference (*p*<0.05). Note: * represents the comparison with that before treatment, *p<0.05; and # represents the comparison with that of group A after treatment, #*p*<0.05.

**Figure 3:**
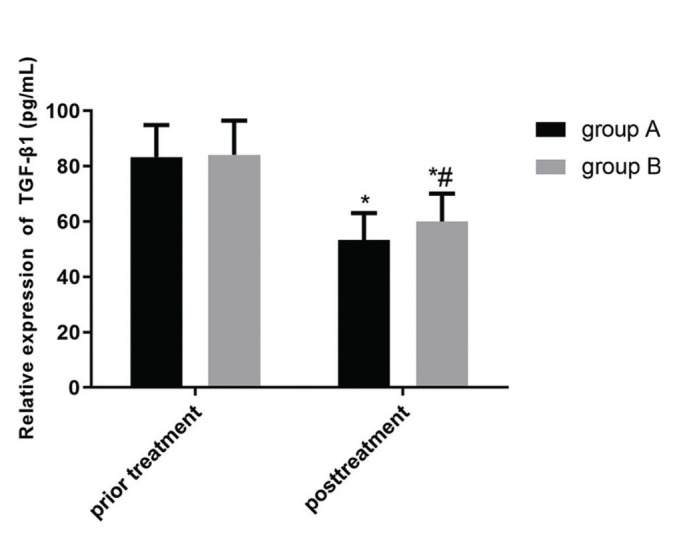
**Comparison of the serum levels of TGF-β1 between the two** **groups.** There is no significant difference in the expression level of serum TGF-β1 before treatment in the two groups (*p*>0.05). The expression level of serum TGF-β1 slightly decreased after treatment in the two groups, which indicated a statistically significant difference (*p*<0.05). Besides, the expression level of serum TGF-β1 in group A is much lower than that in group B after treatment, which indicated a statistically significant difference (*p*<0.05). Note: * represents the comparison with that before treatment, *p<0.05; and # represents the comparison with that of group A after treatment, #*p*<0.05.

Figures 2 and 3 show the changes in expression levels of serum TGF-β1 and cystatin C before and after treatment in the two groups. The expression levels of serum TGF-β1 and cystatin C decreased slightly after treatment, which indicated a statistically significant difference (*p*<0.05). Moreover, the serum expression levels of TGF-β1 and cystatin C in group A were much lower than those in group B after treatment, showing a statistically significant difference (*p*<0.05).

### Comparison of occurrence rates of adverse reactions between the two groups

The incidence of adverse reactions in the two groups was statistically analyzed (Table 3). The incidence of adverse reactions of grade I to II in groups A and B was 21.35% and 11.10%, respectively. No significant difference was observed with regard to the incidence of adverse reactions between the two groups (*p*>0.05). The incidence of adverse reactions of grade III-IV in groups A and B was 4.26% and 2.56%, respectively, with no significant differences between the two groups (*p*>0.05). After the symptomatic treatment, all adverse reactions were alleviated.

### Correlative analysis of serum TGF-β1 and cystatin C levels

**Figure 4:**
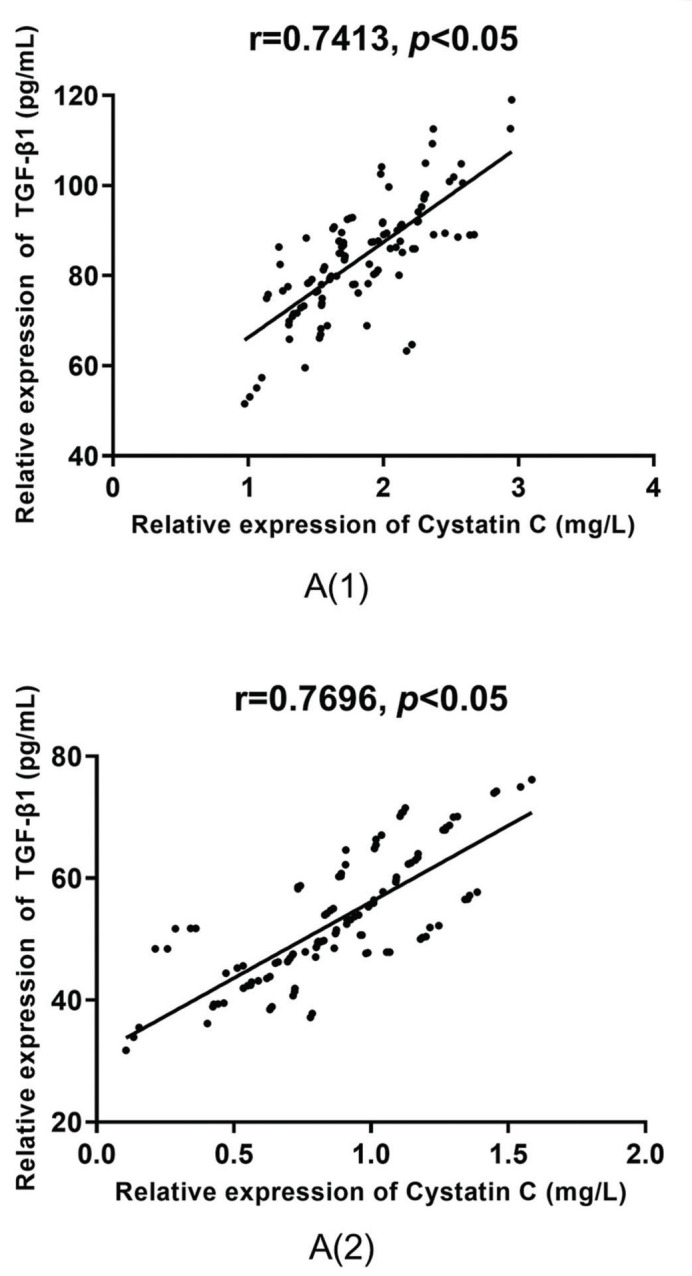
**Correlative analysis of the serum levels of TGF-β1 and cystatin C in group A.** According to the Pearson correlation coefficient analysis, there is a positive correlation between the serum levels of TGF-β1 and cystatin C before and after treatment in group A (r=0.7413 *versus* r=0.7696, *p*<0.05).

**Figure 5:**
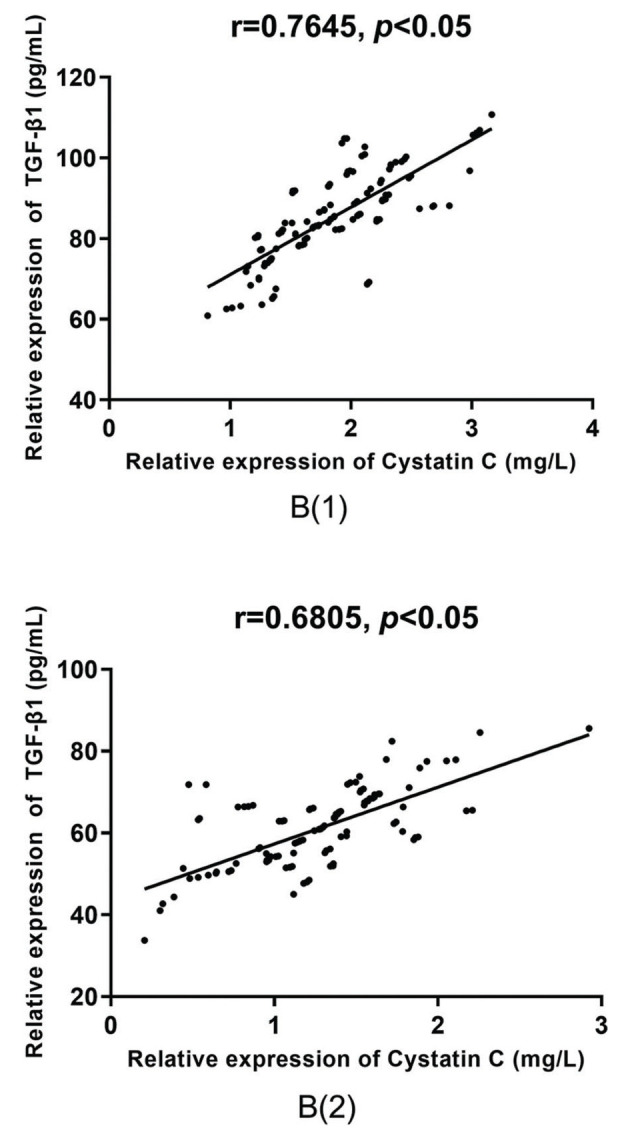
**Correlative analysis of serum TGF-β1 and cystatin C in group B.** According to the Pearson correlation coefficient analysis, there is a positive correlation between the serum levels of TGF-β1 and cystatin C before and after treatment in group B (r=0.7645 *versus* r=0.6805, *p*<0.05).

According to the Pearson correlation coefficient analysis, there was a positive correlation between the serum levels of TGF-β1 and cystatin C in group A (r=0.7413 *versus* r=0.7696, *p*<0.05), as shown in Figure 4, and in group B (r=0.7645 *versus* r=0.6805, *p*<0.05), as shown in Figure 5, before and after treatment.

## DISCUSSION

The symptoms of patients with LN primarily include hematuria, proteinuria, and renal dysfunction (16). In LN, glomerular injury is mainly related to the formation of immune deposits *in situ*; subsequently, an inflammatory reaction is induced via activation of adhesion molecules in the endothelia through immune deposition, thereby leading to the aggregation of proinflammatory cells (17-20). Patients with LN require a treatment that combines various immune agents during a long period (43). The current clinical treatment of LN utilizes cyclophosphamide and glucocorticoid for the reduction of renal injury and improvement of prognosis, to a certain extent; however, the long-term use of these drugs leads to gonadoinhibitory effects and infection, which limit its clinical application. Therefore, it is a controversial topic to seek for a highly efficient and safe therapeutic scheme in clinical research (21,22). Some studies have indicated that mycophenolate mofetil can inhibit the responses of cellular and humoral immunity and selectively inhibit the classical synthetic route of guanine in lymphocytes (23). Cyclophosphamide, a common immunosuppressor with long-term immunosuppressive action and poor anti-inflammatory action, can rapidly reduce the serum immunoglobulin level via reduction of immunoglobulin expression (24). Tacrolimus can interfere with the transcription of cellular factors and activation of T cells by inhibiting calcineurin (25). Compared with cyclosporin A, the inhibitory effect of tarcomus on humoral immunity and cellular immunity is 10~100 times, which can inhibit the expression of interleukin-2 receptor and the production of lymphoid factors such as ?-interferon, interleukin-3 and interleukin-2 (15).

TGF-β1, which is highly expressed in renal tissues, is a fibrogenic factor that helps promote the synthesis and aggregation of collagen and other extracellular matrices during fibrosis, thereby aggravating glomerulosclerosis and renal interstitial fibrosis, and thus, accelerating the development of chronic nephrosis, finally leading to end-stage renal failure (24,26). Cystatin C is a housekeeping gene without histological specificity but with stable expression and transcription in most karyocytes (27). As an endogenous marker, cystatin C can effectively reflect changes in the glomerular filtration rate (GFR) and has a good effect in physiological conditions, and its concentration mainly depends on the GFR. Besides, it can freely pass through the basilar membrane and is completely reabsorbed in the proximal convoluted tubule, without entering blood circulation. Cystatin C, which is removed via the kidney, is not discharged through the renal tubule (28,29).

Based on the therapeutic effects observed in the two groups in this study, the total effective rate of group A was much higher than that of group B. Lee et al. (30) evaluated the comparative efficacy and safety of tacrolimus, mycophenolate mofetil, and cyclophosphamide in the inductive treatment of LN and found that the total effective rate of tacrolimus was much higher than that of mycophenolate mofetil and cyclophosphamide and that tacrolimus most likely reduced the risk of severe infection.

Furthermore, some studies have reported that compared with the intravenous injection of cyclophosphamide, the multi-targeted therapy (tacrolimus and tacrolimus combined with glucocorticoid) had a better therapeutic effect in the inductive treatment of LN in an Asian population (31). In this study, two combination treatments were compared, and the therapeutic effect of mycophenolate mofetil combined with tacrolimus was better than that of mycophenolate mofetil combined with cyclophosphamide in LN. Some studies showed that the multi-targeted therapy of tacrolimus combined with mycophenolate mofetil could be used to treat patients with severe crescent LN, whose symptoms were completely alleviated within 6 months (30). The time of effectiveness and time of effect validity of group A were much lower than those of group B. This indicated that tacrolimus combined with mycophenolate mofetil had a better and faster therapeutic effect. Some existing studies show that cyclophosphamide can effectively reduce the expression levels of serum cystatin C in LN patients, with a better therapeutic effect and higher safety (32). However, there are few studies on the influence of mycophenolate mofetil combined with tacrolimus and of mycophenolate mofetil combined with cyclophosphamide on serum TGF-β1 and cystatin C in patients with LN. In this study, changes in the expression levels of serum TGF-β1 and cystatin C, before and after treatment, in the two groups were recorded. There were no significant differences in the expression levels of serum TGF-β1 and cystatin C in the two groups before treatment. The expression levels of serum TGF-β1 and cystatin C slightly decreased after treatment in the two groups, and those of group A were much lower than those of group B, after treatment. These results indicate that a modulation of the expression of TGF-β1 and cystatin C can improve the conditions of patients with LN and that tacrolimus demonstrated more a significant effect compared with cyclophosphamide. According to previous studies, TGF-β1 is one of the factors modulating immunoregulation. TGF-β1 is produced via IL-2, inhibits the proliferation of T cells, and reduces the levels of IL-6, IL-1, and TNF-α in macrophages (33) by signaling CD4+ cells to divide into Th1, Th2, Th17, and CD8+ cytotoxic T cells. Cystatin, a member of the housekeeping genes, is expressed in all karyocytes and is influenced by acute changes in kidney function (34). However, only a few reports on the regulation mechanism of cystatin C in LN have been published. As per previous reports, the adverse drug reactions caused by tacrolimus were mostly reversible and could be improved by reducing the drug dose (35). There was no significant difference in the occurrence rates of adverse reactions in the two groups. Moreover, the degree of adverse reactions was mild in the two groups; thus, the symptoms could be alleviated after symptomatic treatment. Wu et al. (36) compared the occurrence rates of adverse reactions between tacrolimus combined with prednisone tablets and cyclophosphamide combined with prednisone tablets in the treatment of LN and found that the occurrence rate of adverse reactions of tacrolimus was much lower than that of cyclophosphamide, with a statistically significant difference. Jiang et al. (37) found that there was no significant difference in the occurrence rates of adverse reactions between mycophenolate mofetil combined with tacrolimus and intravenous pulse cyclophosphamide therapy. These inconsistent results may be due to differences in sample size, as well as in the medical technologies used; however, all the results indicated that the multi-targeted therapy of tacrolimus combined with mycophenolate mofetil was safe and reliable, with a low occurrence rate of adverse reactions. Some studies showed that TGF-β1 was highly expressed in LN (38). Furthermore, Chew et al. (39) reported that cystatin C was highly expressed in patients with a medical history of LN. Cystatin C can be regarded as an important marker in LN (40). From the Pearson correlation coefficient analysis, a positive correlation between the serum levels of TGF-β1 and cystatin C before and after treatment in the two groups was obtained. Zhao et al. (41) also found that TGF-β1 was positively correlated with serum cystatin C in children with Henoch-Schönlein purpura nephritis. Some studies have reported that TGF-β1 can inhibit the degradation of the extracellular matrix by cathepsins by up-regulating the secretion of cystatin C (42). However, presently, there are few correlative studies on TGF-β1 and cystatin C in LN; thus, it is necessary to conduct further research on this topic.

In this study, the therapeutic effects of mycophenolate mofetil in combination with tacrolimus and those of mycophenolate mofetil in combination with cyclophosphamide were analyzed and their influence on serum TGF-β1 and cystatin C levels was comprehensively discussed. The prognosis of patients with LN and the corresponding influential factors should be evaluated in future to determine the functional mechanisms underlying the therapeutic effects of the combination of mycophenolate mofetil with tacrolimus in LN patients, and thus, provide further insights into the pathogenesis, clinical diagnosis, and treatment of LN.

Therefore, mycophenolate mofetil in combination with tacrolimus reduced serum TGF-β1 and cystatin C levels in patients with LN and showed a better and safer clinical effect than that of mycophenolate mofetil in combination with cyclophosphamide.

## AUTHOR CONTRIBUTIONS

Zhang X and Zhang Z, conceived the study and designed the experiments. Zhang X, Liu P, and Zhang Z, contributed to the data collection, performed the data analysis, and interpreted the results. Zhang X wrote the manuscript. Zhang Z contributed to the critical revision of the article. All authors read and approved the final manuscript.

## Figures and Tables

**Table 1:** Comparison of the general data in the two groups (x±SD)/[n (%)].

	Group A (n=117)	Group B (n=117)	X^2^/t	*P*-value
Sex			0.436	0.509
Male	53 (45.30)	48 (41.03)		
Age (years)			0.285	0.594
<30	45 (38.46)	49 (41.88)		
≥30	72 (61.54)	68 (58.12)		
BMI (kg/m^2^)	22.15±3.07	22.63±3.11	1.188	0.236
Average course of disease (months)	11.65±4.57	11.32±4.21	0.575	0.566
Smoking habit			0.160	0.690
Yes	46 (39.32)	49 (41.88)		
Drinking habit			0.073	0.787
Yes	74 (63.25)	72 (61.54)		
Blood platelets (×10^4^/μL)	24.76±9.68	26.08±10.12	1.020	0.309
Hemoglobin (g/L)	96.79±22.79	97.21±24.97	0.134	0.893
Pathological type			0.159	0.924
Type III	29 (24.79)	28 (23.93)		
Type IV	53 (45.30)	56 (47.86)		
Type V	35 (29.91)	33 (28.21)		
Systolic Blood Pressure (SBP) (mmHg)	126.28±12.01	124.19±11.78	1.344	0.180
Diastolic Blood Pressure (DBP) (mmHg)	71.09±8.76	72.01±7.68	0.854	0.394
Chronicity index (mg/dL)	1.67±0.78	1.59±0.59	0.885	0.377
Serum urea (mg/dL)	60.28±40.19	53.28±37.39	1.379	0.169
Urinary 24-h protein excretion (gm)	3.71±0.37	3.68±0.33	0.655	0.513
C3 (g/L)	0.78±0.17	0.75±0.13	1.516	0.131
eGFR (mL/min)			0.819	0.664
<60	42 (35.90)	38 (32.48)		
60-90	57 (48.72)	56 (47.86)		
>90	18 (15.38)	23 (19.66)		
Positive anti-dsDNA, n (%)	101 (86.32)	105 (89.74)	0.649	0.420

**Table 2:** Comparison of the therapeutic effects between the two groups [n (%)].

	CR	PR	Invalid	Total effective rate
Group A (n=117)	59 (50.43)	47 (40.17)	11 (9.40)	90.60%
Group B (n=117)	52 (44.44)	42 (35.90)	23 (19.66)	80.34%
X^2^				4.880
*p*				0.027

**Table 3:** of the occurrence rates of adverse reactions between the two groups [n (%)].

	I~II								
	Nausea and vomiting	Gastrointestinal reaction	Erythra	Pulmonary infection	Aleucocytosis	Lipsotrichia	Kidney injury	Nerve damage	Incidence of adverse reactions
Group A (n=117)	8 (6.84)	5 (4.27)	5 (4.27)	2 (1.71)	1 (0.85)	1 (0.85)	2 (1.71)	1 (0.85)	21.35%
Group B (n=117)	6 (5.13)	2 (1.71)	3 (2.56)	1 (0.85)	0 (0.00)	0 (0.00)	1 (0.85)	0 (0.00)	11.10%
X^2^									3.720
*p*									0.054

	III~IV								
	Nausea and vomiting	Gastrointestinal reaction	Erythra	Pulmonary infection	Aleucocytosis	Lipsotrichia	Kidney injury	Nerve damage	Incidence of adverse reactions
Group A (n=117)	3 (2.56)	1 (0.85)	0 (0.00)	0 (0.00)	0 (0.00)	0 (0.00)	1 (0.85)	0 (0.00)	4.26%
Group B (n=117)	1 (0.85)	2 (1.71)	0 (0.00)	0 (0.00)	0 (0.00)	0 (0.00)	0 (0.00)	0 (0.00)	2.56%
X^2^									0.687
*p*									0.407
